# Evaluation of the intrinsic and extrinsic ecological effects on the helminth communities of *Didelphis albiventris* (Didelphimorphia, Didelphidae) in Atlantic Forest areas

**DOI:** 10.1590/S1984-29612026036

**Published:** 2026-08-03

**Authors:** Filipe Andrade Rangel, Thiago dos Santos Cardoso, Bruna Silva Cirino, Sócrates Fraga da Costa-Neto, Camila dos Santos Lucio, Roberto do Val Vilela, Arnaldo Maldonado-Júnior, Rosana Gentile

**Affiliations:** 1 Instituto Oswaldo Cruz, Fundação Oswaldo Cruz – FIOCRUZ, Laboratório de Biologia e Parasitologia de Mamíferos Silvestres Reservatórios, Rio de Janeiro, RJ, Brasil; 2 Instituto Oswaldo Cruz, Fundação Oswaldo Cruz – FIOCRUZ, Programa de Pós-graduação em Biodiversidade e Saúde, Rio de Janeiro, RJ, Brasil; 3 Fiocruz Mata Atlântica, Fundação Oswaldo Cruz – FIOCRUZ, Rio de Janeiro, RJ, Brasil

**Keywords:** Ecology, metacommunities, opossum, parasitism, Ecologia, metacommunidades, gambá, parasitismo

## Abstract

The white-eared opossum *Didelphis albiventris* is widely distributed throughout Brazil and hosts several parasite species. This study aimed to analyze the influence of intrinsic (sex and age of hosts) and extrinsic (locality and type of land use) factors on the helminth communities of the white-eared opossum in areas of the Atlantic Forest. Helminth samples from 93 *D. albiventris* specimens from six different localities were used: Belo Horizonte, MG; Curitiba and Guaíra, PR; Porto Alegre, RS; and Mamanguape and Santa Rita, PB. Fourteen species of helminths were identified: *Aspidodera raillieti*, *Cruzia tentaculata*, *Turgida turgida*, *Viannaia hamata*, *Rhopalias coronatus*, *Rhopalias horridus*, *Duboisiella proloba*, *Travassostrongylus orloffi*, *Travassostrongylus sextus*, *Brachylaima advena*, *Plagiorchis didelphidis*, *Trichuris minuta*, *Trichuris didelphis*, and *Oligacanthorhynchus microcephalus*. Female hosts and adult hosts had greater helminth species richness and abundance. The helminth species richness and abundance in *D. albiventris* were influenced by intrinsic (sex and age of the host) and extrinsic (locality and land use) factors with the locality being the main determinant. The structure of the helminth infracommunities varied spatially, with greater differences with increasing geographic distance, highlighting the complexity of host‒parasite interactions in wildlife.

## Resumo

O gambá-de-orelha-branca – *Didelphis albiventris* – possui ampla distribuição no Brasil e hospeda diversas espécies de parasitos. Este estudo teve como objetivo analisar a influência de fatores intrínsecos (sexo e idade dos hospedeiros) e extrínsecos (localidade e tipo de uso da terra) sobre as comunidades de helmintos do gambá-de-orelha-branca em áreas da Mata Atlântica. Foram utilizadas amostras de helmintos de 93 espécimes de *D. albiventris* provenientes de seis localidades diferentes: Belo Horizonte, MG; Curitiba e Guaíra, PR; Porto Alegre, RS; e Mamanguape e Santa Rita, PB. Foram identificadas quatorze espécies de helmintos, sendo elas: *Aspidodera raillieti*, *Cruzia tentaculata, Turgida turgida*, *Viannaia hamata*, *Rhopalias coronatus*, *Rhopalias horridus*, *Duboisiella proloba*, *Travassostrongylus orloffi*, *Travassostrongylus sextus*, *Brachylaima advena*, *Plagiorchis didelphidis*, *Trichuris minuta*, *Trichuris didelphis* e *Oligacanthorhynchus microcephalus*. Hospedeiros fêmeas e hospedeiros adultos apresentaram maior riqueza e abundância de espécies de helmintos. A riqueza e a abundância de espécies de helmintos em *D. albiventris* foram influenciadas por fatores intrínsecos (sexo e idade do hospedeiro) e extrínsecos (localidade e uso da terra), sendo a localidade o principal determinante. A estrutura das infracomunidades de helmintos variou espacialmente, apresentando maiores diferenças com o aumento da distância geográfica, evidenciando a complexidade das interações parasito-hospedeiro na natureza.

## Introduction

Parasitism plays an important role in the structuring of host communities and is fundamental to the dynamics of natural ecosystems. Studies of parasite diversity and their transmission cycles are essential for understanding the ecology of interactions and the ecology of communities. Additionally, parasites play a prominent role as indicators of ecosystem health (Marcogliese, 2005; Brandão et al., 2009; Tompkins et al., 2011). Despite its importance and due to this recognition only in recent decades (Poulin, 2007), there is still a large gap in the knowledge of parasite diversity and the ecological factors that influence parasite occurrence and distribution (Dallas & Presley, 2014; Preisser, 2019; Cirino et al., 2022).

Spatial variation in the composition and structure of wildlife parasite communities can be assessed along an environmental gradient (Dallas & Presley, 2014), in which intrinsic host conditions such as sex, age, body size, phylogeny, and the immune response to infection influence this gradient. Thus, the dynamics of the transmission of parasites in the environment become better understood when larger spatial scales are considered than those usually employed in local studies (Fernandes et al., 2014; Suzán et al., 2015).

Small, non-flying mammals, such as marsupials from the Neotropical region, constitute groups of special interest for studies of parasitism, since many species are known to act as natural reservoirs of parasites with zoonotic potential (Bitencourt & Bezerra, 2022). Helminths are particularly relevant in this context because of their high species diversity, broad host range, and importance to public health (Han et al., 2015, 2016). In addition, helminths are good models for the study of host‒parasite ecological interactions, mainly because of their dispersal potential and the relative ease with which their abundance can be estimated (Poulin, 2007).

Species of opossums have been reported to host several helminth species, including species of medical and veterinary interest (Bezerra-Santos et al., 2021). The white-eared opossum *Didelphis albiventris* is a species that occurs in different environments, including degraded and conserved areas of the Atlantic Forest, Caatinga, Cerrado, Pantanal, Pampa, and their ecotones (Faria et al., 2019; Cáceres et al., 2023). *Didelphis albiventris* occurs in open and deciduous forest types from northeastern Brazil to mid-Argentina. In the Atlantic Forest, its occurrence is largely restricted to ecotonal zones or areas with a more open vegetation structure. It is replaced in wetter Atlantic and Araucaria forests by *Didelphis aurita*, in Amazonian forests by *Didelphis marsupialis*, and on slopes of the Andes by *Didelphis pernigra* (Gardner, 2008). Sympatry with *D. aurita* or *D. marsupialis* occurs only in areas disturbed by humans (Varejão & Valle 1982, Gardner, 2008). This marsupial has been used as a model for different studies aimed at understanding the ecology of its parasite fauna (Quintão e Silva & Costa, 1999; Müller, 2005; Pinto et al., 2014; Ramos et al., 2016; Zabott et al., 2017; Cirino et al., 2022, 2025; Illia et al., 2024; Hartmann et al., 2025). However, except for the studies by Cirino et al. (2022, 2025), most of the studies mentioned above focused on understanding local factors and did not evaluate spatial effects on the helminth community.

This study aimed to describe helminth communities and investigate intrinsic and extrinsic host-related factors influencing helminth species richness and abundance in *D. albiventris* in preserved and disturbed localities of the Brazilian Atlantic Forest. The central hypothesis of this study was that the processes responsible for the structure of helminth communities in the opossum *D. albiventris* are determined by host attributes, such as sex and age, as well as by extrinsic factors, including geographic distance and local environmental effects.

## Material and Methods

### Study areas

**Figure 1:**
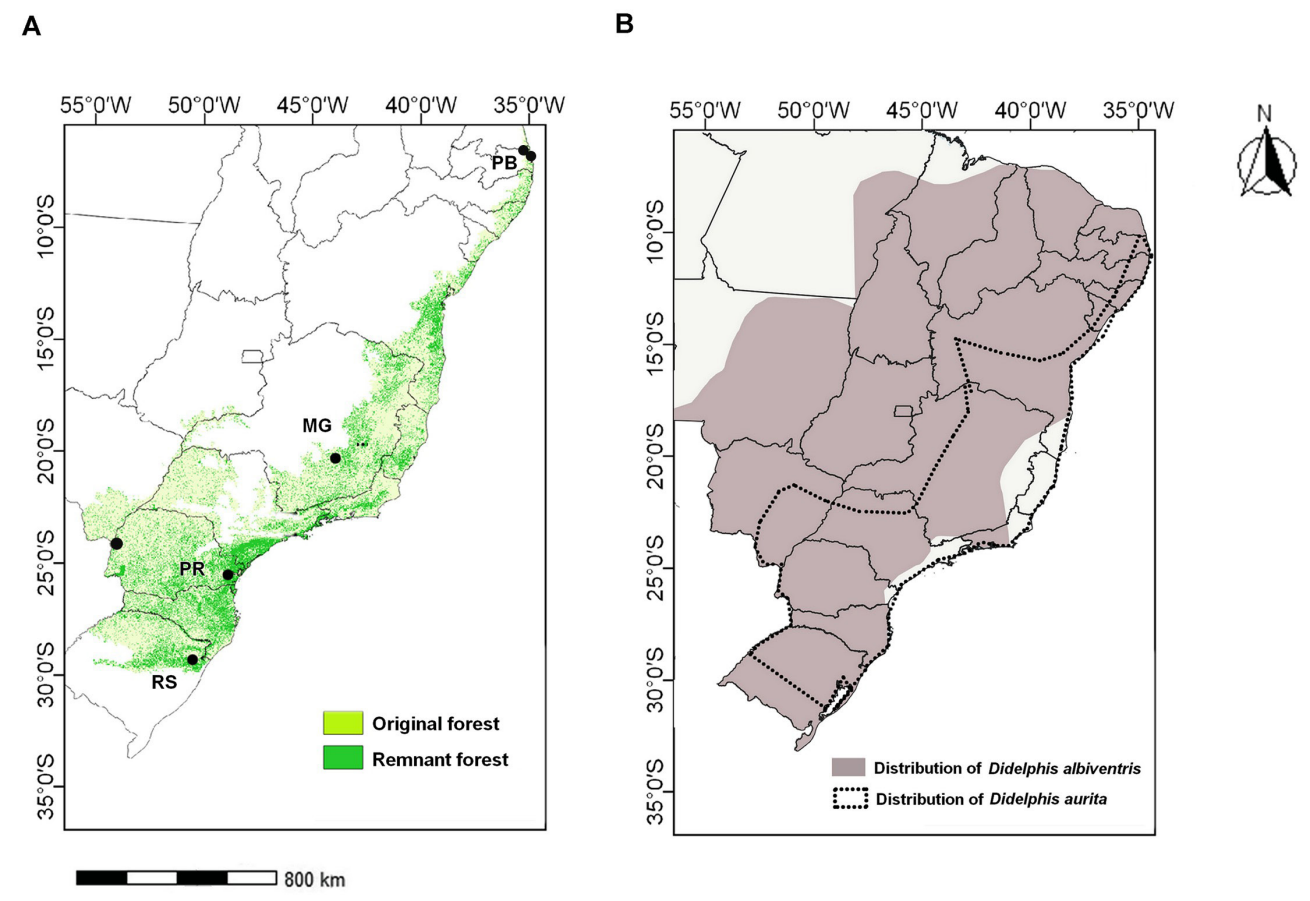
Localities and municipalities where *Didelphis albiventris* was collected along the Brazilian Atlantic Forest (A), and distribution map of *D. albiventris* and *D. aurita* in Brazil (B).

The hosts were collected from six Brazilian localities in the municipalities of Porto Alegre (RS), Curitiba and Guaíra (PR) in the South Region, Belo Horizonte (MG) in the Southeast Region, and Santa Rita and Mamanguape (PB) in the Northeast Region (Figure 1A). The sampling in Porto Alegre, the capital of the state of Rio Grande do Sul (RS), was performed in peri-domicile areas, with transects installed in the backyards of the residents near forest fragments. The areas included the Vale Campus of the Federal University of Rio Grande do Sul (30°4'14.73''S, 51°7'16.35''W), the Vila Laranjeira community (30°2'57.82''S, 51°7'51.65''W), Santana Hill (30°01'59.1"S, 51°07'39.4"W), and Police Hill (30°03'12.0"S, 51°08'55.0" W).

The samplings in Curitiba, the capital of the state of Paraná (PR), were performed at urban parks within the Metropolitan Region of Curitiba: Parque Náutico Municipal de Curitiba (25°31'02.3"S, 49°12'40.4"W), Zoológico Municipal de Curitiba (25°33'09"S, 49°14'07"W), and Jardim Botânico Municipal de Curitiba (25°26'30.1"S, 49°14'14.1"W).

In Guaíra, PR, sampling occurred at Debus Farm, a peri-urban locality, which is part of an area of great environmental relevance near Ilha Grande National Park (24°03'00"S, 54°16'00"W). Additional samples were obtained from Vila Maldiva (24°05'21"S, 54°15'10"W), a peri-urban community located on the banks of the Paraná River adjacent to Ilha Grande National Park.

In Belo Horizonte, the capital of the state of Minas Gerais (MG), four different sites were sampled within the Metropolitan Region: Cidade Administrativa Presidente Tancredo de Almeida Neves (19°47'13"S, 47°57'02"W), Parque Ecológico Francisco Lins do Rêgo – known as Parque Ecológico da Pampulha (19°51'23"S, 43°59'47"W), Comunidade Quilombola de Mangueiras (19°49'28"S, 43°54'16"W), and Parque Olinto Marinho Couto – formerly Parque Bosque São Bento II (19°57'10"S, 43°57'27"W).

The samples from Santa Rita and Mamanguape, state of Paraíba (PB), were concentrated in preserved areas of the Atlantic Forest. In Santa Rita, the collections occurred in the Gargaú Natural Heritage Private Reserve (RPPN), also known as Mata de Santana (7°00'44” S, 34°57'25” W), which has forest fragments in various stages of ecological succession. In Mamanguape, collections were made in the Guaribas Biological Reserve (6°45'32"S, 35'12'51"W), one of the last remnants of the Atlantic Forest in Northeast Brazil.

### Methods of helminth collection and fixation

The collections in Curitiba, PR, were performed in April 2019 for four consecutive nights. In Porto Alegre, RS, samples were collected in April 2018; in Guaíra, PR, samples were collected in April 2024; and in Belo Horizonte, MG, samples were collected in September 2019, all for five consecutive nights. In Santa Rita, PB, the collections took place between the end of August and the beginning of September 2017 for six consecutive nights; in Mamanguape, PB, the collections were performed in June 2014 and April 2015 for ten consecutive nights. Captures were made using Tomahawk Live Traps, Model 201 (dimensions: 40.64 × 12.7 × 12.7 cm; USA), on transects of 15 points spaced 20 m apart.

The captured animals were anesthetized and euthanized for the collection of helminths and other samples. The procedures followed the protocols of the Laboratório de Biologia e Parasitologia de Mamíferos Silvestres Reservatórios (LABPMR), Instituto Oswaldo Cruz, FIOCRUZ (IOC/FIOCRUZ) (D’Andrea et al., 2021). The captures were performed under collection licenses from the Brazilian Government’s Chico Mendes Institute for Biodiversity and Conservation (ICMBIO, license 13373). All procedures followed the guidelines for the capture, handling, and care of animals of the Ethical Committee on Animal Use of the Oswaldo Cruz Foundation (CEUA, licenses LW-39/14, L-36/18, and L-036/2018-A1) and followed biosafety protocols for the capture and handling of wild animals (Lemos & D'Andrea, 2014). The collected animals were preserved by taxidermy, and their skeletons were prepared and deposited as voucher material in the Integrated Collection of Wild and Reservoir Mammals (COLMASTO) of the Oswaldo Cruz Institute. The identification of the animals was performed based on external morphology by the National Reference Laboratory for Taxonomy and Diagnosis of Wild Reservoirs of Leishmaniasis of the Oswaldo Cruz Institute.

The animals were dissected, and their organs were individually separated, placed in Petri dishes and immersed in a saline solution (0.85% NaCl). The trachea, lungs, esophagus, heart, kidneys, liver, pancreas, spleen, small intestine, large intestine, cecum, mesenteric veins, reproductive system and body cavities were examined for the presence of helminths. The helminths found were placed in Petri dishes with saline solution (0.85% NaCl). Specimens larger than 0.5 mm were transferred to a Petri dish using fine forceps and a soft brush. Specimens smaller than 0.5 mm were visualized and collected with the aid of a stereoscopic microscope, and a fine brush was used to remove debris from the collected specimens. Some nematode specimens were fixed in AFA (93 parts of 70% ethanol, 5 parts of 0.4% formaldehyde, and 2 parts of 100% acetic acid) and heated to 65°C, whereas others were kept in 70% ethanol for molecular genetic studies.

### Helminth identification methods

Helminth species identification was performed using an Axio Scope microscope. A1 – Zeiss coupled to an Axio Cam MRc digital camera for photomicrography. The nematodes were diaphanized with lactophenol or 50% glycerol and adjusted between the slide and coverslip for identification at the species level. The trematodes, cestodes and acanthocephalans were stained with Langeron Carmine, differentiated by 0.5% hydrochloric alcohol, dehydrated in an increasing alcoholic series, diaphanized in methyl salicylate, and mounted in Canada Balsam, with permanent preparation (Amato et al., 1991). The helminths were identified to the species level whenever possible and then counted and separated by sex (except in the case of hermaphrodite species). The specific morphological diagnosis features used to identify the specimens followed Vicente et al. (1997) and Anderson et al. (2009) for Nematoda, Travassos et al. (1969) for Trematoda, Gomes (1977) for Cestoda and Acanthocephala, in addition to the species description publications.

### Data analyses

The mean abundance and mean intensity with their standard deviations and the prevalence, with confidence intervals, of each helminth species were calculated for each investigated variable, according to Bush et al. (1997). The mean abundances were calculated by dividing the total number of parasites by the total number of hosts. To calculate the mean intensity, the total number of parasites was divided by the number of infected hosts. For prevalence, the number of infected hosts was divided by the total number of hosts collected and then multiplied by 100. The helminth species richness, represented by the total number of species, and total abundance, represented by the total sum of helminth specimens, were calculated for each infracommunity.

To investigate the factors that influence helminth species richness and helminth abundance, generalized linear models (GLMs) with Gaussian distributions were fitted. All the host specimens analyzed were infected by helminths with high parasite loads, which differs from the common aggregated pattern of most parasites. The distribution of the data was verified by analyzing the distribution of scaled residuals from the global model, according to Hartig (2024), and fitted a Gaussian distribution. The explanatory variables were land use (preserved or peri-urban areas), host sex (male or female), host age (young or adult) and the locality where the hosts were captured (Porto Alegre, Curitiba, Guaíra, Belo Horizonte, Mamanguape and Santa Rita). After the global models were fit, the models were selected based on the corrected Akaike Information Criterion (AICc) to identify which variables best explained the variation in helminth species richness and abundance. The plausible models considered were those with a delta AICc ≤ 2 and greater weight (AICcWt).

The influence of spatial scale on the abundance of each helminth species in the infracommunities was also investigated as follows. First, an abundance matrix of helminth species per infracommunity was constructed, which was transformed by the Hellinger method (Legendre & Gallagher, 2001) to reduce the influence of rare species and make the data suitable for linear methods such as redundancy analysis (RDA). To represent the spatial gradient between the infracommunities, an approach based on distance-derived Moran Eigenvector Maps (dbMEM; Dray et al., 2006) was used to represent spatial patterns at different scales. The geographic coordinates (latitude and longitude) of each location where the infracommunities were sampled were used to calculate a matrix of geodesic distances between the points. This method considers the curvature of the Earth and is more appropriate for spatial data in geographic coordinates (Dray et al., 2012). The dbMEMs represent spatial patterns at multiple scales, ranging from large (first axes) to small (last axes), allowing the identification of broad spatial gradients and local variations. To identify which spatial axes (dbMEM) influenced the species abundance matrix of the infracommunities, forward selection based on the adjusted R^2^ criterion was applied (Blanchet et al., 2008).

All analyses were performed in the R software environment (R Core Team, 2026). For the spatial analyses, the geosphere (Hijmans et al., 2017) and adespatial (Dray et al., 2022) packages were used. Hellinger transformation and forward selection of spatial descriptors (dbMEM) were performed with the vegan package (Oksanen et al., 2025). The GLM was performed with the stats package (R Core Team, 2026), and the adequacy of the models was verified with the DHARMa package (Hartig, 2024).

## Results

Helminths of the phyla Nematoda, Platyhelminthes (classes Cestoda and Trematoda), and Acanthocephala were collected from 93 specimens of *D. albiventris* as follows: 15 from Porto Alegre, 12 from Curitiba, 14 from Guaíra, 11 from Belo Horizonte, 17 from Santa Rita, and 24 from Mamanguape (Table 1). The species found were *Aspidodera raillieti* Travassos, 1913; *Cruzia tentaculata* (Rudolphi 1819) Travassos, 1917; *Turgida turgida* (Rudolphi, 1819) Travassos, 1920; *Viannaia hamata* Travassos, 1914; *Travassostrongylus orloffi* Travassos, 1935; *Travassostrongylus sextus* Freitas, 1937; *Trichuris didelphis* Babero, 1960; *Trichuris minuta* (Rudolphi, 1819); *Brachylaima advena* Dujardin, 1843; *Plagiorchis didelphidis* (Parona, 1896) Stossich, 1904; *Rhopalias coronatus* (Rudolphi, 1819) Stiles & Hassall, 1898; *Rhopalias horridus* (Diesing, 1850) Stiles & Hassall, 1898; *Duboisiella proloba* Baer, 1938; and *Oligacanthorhynchus microcephalus* (Rudolphi, 1819) Schmidt, 1972.

**Table 1:** Total number of *Didelphis albiventris* hosts (n=93) and number of helminths recovered for each species, by locality, in Porto Alegre, Curitiba, Guaíra, Belo Horizonte, Mamanguape and Santa Rita, Brazil.

**Phylum/Class**	**Localities**					
	**Porto Alegre (n=15)**	**Curitiba (n=12)**	**Guaíra (n = 14)**	**Belo Horizonte(n=11)**	**Mamanguape (n=24)**	**Santa Rita(n=17)**
**Phylum Nematoda Rudolphi, 1808**						
***Aspidodera raillieti***	353	319	76	146	474	1199
***Cruzia tentaculate***	3701	561	349	1923	2310	7126
***Travassostrongylus orloffi***	32	5	0	0	35	82
***Travassostrongylus sextus***	0	5	0	0	0	0
***Trichuris didelphis***	21	0	14	0	40	92
***Trichuris minuta***	17	3	0	0	14	196
***Trichuris* sp.**	0	0	0	5	0	0
***Turgida turgida***	74	89	61	203	218	193
***Viannaia hamata***	1025	2	0	18	1561	2353
***Viannaia* sp.**	0	0	0	0	21	86
**Phylum Platyhelminthes Minot, 1876**						
Class Trematoda						
***Brachylaima advena***	3	94	0	0	9	0
***Duboisiella proloba***	0	0	0	61	0	0
***Plagiorchis didelphidis***	0	79	0	0	0	0
***Rhopalias coronatus***	0	24	0	38	0	0
***Rhopalias horridus***	44	26	0	152	0	0
**Class Cestoda**						
**Cestoda**	0	0	0	0	5	0
**Phylum Acanthocephala Rudolphi, 1802**						
***Oligacanthorhynchus microcephalus***	0	0	48	0	0	0

Specimens of *Viannaia*sp. were found in the localities of Mamanguape and Santa Rita. These specimens are distinct from *Viannaia hamata* and from the others 18 species of this genus, thus, probably representing a new species, which will be described elsewhere. *Trichuris*sp. were found in the locality of Belo Horizonte and they could not be identified at the species level because there were only female individuals. Thus, this morphotype was the only one not included in the GLM and dbMEM analyses. Cestode individuals were found in the locality of Mamanguape and could not be identified because of its poor state of conservation (Table 1).

The mean abundances (Tables 2, 3 and 4), mean intensities (Tables 5, 6 and 7) and prevalence rates (Tables 8, 9 and 10) were calculated for the helminth species recovered from each location, as well as for host attributes such as sex (male/female) and age (young/adult). The species with the greatest abundance among all the infracommunities were *C. tentaculata* (16,106 specimens), *V. hamata* (5,796 specimens), *A. raillieti* (2,567 specimens), and *Tu. turgida* (1,071 specimens) (Tables 2, 3 and 4). These four species were also the most prevalent, with prevalence rates and confidence intervals of 81.72 [72.35 – 88.98], 66.67 [56.13 – 76.11], 56.99 [46.31 – 67.22], and 83.87 [74.80 – 90.68], respectively. *C. tentaculata* and *V. hamata* also presented the highest mean intensities with high standard deviations (211.92 ± 233.63 and 109.36 ± 128.65, respectively).

**Table 2:** Mean abundance of helminths ± standard deviations and range size in parenthesis in relation to host sex and age recovered from *Didelphis albiventris* by locality in Porto Alegre, Curitiba, Guaíra, Belo Horizonte, Mamanguape and Santa Rita, Brazil.

**Parameters/Species**	***Aspidodera raillieti***	***Cruzia tentaculata***	***Travassostrongylus orloffi***	***Travassostrongylus sextus***	***Trichuris didelphis***	***Trichuris minuta***	***Trichuris* sp.**
**Mean AbundanceTotal (N = 93)**	27.60 ± 54.14 (0 – 401)	173.18 ± 226.45 (0 – 1.125)	1.38 ± 4.23 (0 – 28)	0.05 ± 0.52 (0 – 5)	1.80 ± 7.21 (0 – 60)	2.47 ± .8.98 (0 – 60)	0.05 ± 0.43 (0 – 4)
**Male(N = 56)**	26.38 ± 76.11 (0 – 401)	126.66 ± 164.90 (0 – 685)	0.98 ± 3.51 (0 – 15)	0.09 ± 0.67 (0 – 5)	1.57 ± 10.79 (0 – 60)	2.18 ± 10.33 (0 – 41)	0.09 ± 0.55 (0 – 4)
**Female (N = 34)**	28.03 ± 39.97 (0 – 197)	239.12 ± 283.99 (0 – 1125)	2.12 ± 5.76 (0 – 28)	0	2.00 ± 4.82 (0 – 19)	3.18 ± 10.57 (0 – 60)	0
**Young (N = 36)**	13.97 ± 15.56 (0 – 71)	104.69 ± 137.34 (0 – 617)	3.14 ± 9.06 (0 – 50)	0.14 ± 0.83 (0 – 5)	3.72 ± 10.98 (0 – 60)	1.69 ± 6.04.(0 – 34)	0
**Adult (N = 52)**	30.38 ± 66.70 (0 – 401)	220.40 ± 269.15 (0 – 1.125)	0.58 ± 2.54 (0 – 16)	0	0.63 ± 2.60 (0 – 14)	.2.35 ± 9.51 (0 – 60)	0.10 ± 0.57 (0 – 4)
**Belo Horizonte (N = 11)**	13.27 ± 37.80 (0 – 127)	174.82 ± 240.29 (0 – 590)	0	0	0	0	0.45 ± 1.21 (0 – 4)
**Curitiba (N = 12)**	26.58 ± 26.80 (0 – 91)	47.75 ± 53.16 (0 – 159)	0.42 ± 1.16 (0 – 4)	0.05 ± 0.52 (0 – 5)	0	0.25 ± 0.87 (0 – 3)	0
**Porto Alegre (N = 15)**	23.53 ± 27.15 (0 – 71)	246.73 ± 156.11 (24 – 531)	2.13 ± 4.75 (0 – 16)	0	1.40 ± 3.68 (0 – 13)	1.13 ± 3.07 (0 – 12)	0
**Mamanguape (N = 24)**	19.75 ± 35.00 (0 – 125)	96.25 ± 121.22 (0 – 523)	1.46 ± 3.49 (0 – 13)	0	1.67 ± 4.75 (0 – 21)	0.58 ± 1.98 (0 – 4)	0
**Santa Rita (N = 17)**	70.53 ± 100.44 (0 – 401)	419.18 ± 320.69 (0 – 1.125)	4.82 ± 7.52 (0 – 28)	0	5.41 ± 14.98 (0 – 60)	7.35 ± .18.49 (0 – 60)	0
**Guaíra (N = 14)**	5.43 ± 20.31 (0 – 76)	33.79 ± 74.26 (0 – 250)	0	0	1.00 ± 3.74 (0 – 14)	0	0

N = number of hosts examined.

**Table 3:** Mean abundance of helminths ± standard deviations and range size in parenthesis in relation to host sex and age recovered from *Didelphis albiventris* by locality in Porto Alegre, Curitiba, Guaíra, Belo Horizonte, Mamanguape and Santa Rita, Brazil.

**Parameters/Species**	***Turgida turgida***	***Viannaia hamata***	***Viannaia* sp.**	***Brachylaima advena***	***Duboisiella proloba***	***Plagiorchis didelphidis***	***Rhopalias coronatus***
**Mean Abundance Total (N =93)**	11.52 ± 18.70 (0 – 135)	62.32 ± 110.51 (0 – 568)	.1.15 ± 9.11.(0 – 86)	0.30 ± 1.67 (0 – 15)	0.66 ± 4.27 (0 – 38)	0.85 ± 8.09 (0 – 78)	0.67 ± 4.02 (0 – 30)	
**Male (N= 56)**	9.25 ± 7.90 (0 – 45)	48.96 ± 98.60 (0 – 399)	1.91 ± 15.00 (0 – 86)	0.11 ± 0.17 (0 – 3)	1.09 ± 5.48 (0 – 38)	0	0.68 ± 4.10 (0 – 30)	
**Female (N = 34)**	15.76 ± 27.35 (0 – 135)	82.32 ± 142.61 (0 – 568)	0	0.65 ± 2.70 (0 – 15)	0	0.03 ± 0.17 (0 – 1)	0.71 ± 4.12 (0 – 26)	
**Young (N = 36)**	4.25 ± 4.99 (0 – 22)	63.14 ± 82.61 (0 – 330)	0.06 ± 0.33 (0 – 2)	0.67 ± 2.64 (0 – 15)	0	2.17 ± 13.00 (0 – 78)	0	
**Adult (N = 52)**	15.35 ± 20.82 (0 – 135)	52.83 ± 119. 26 (0 – 568)	.2.02 ± 12.16.(0 – 86)	0.08 ± 0.33 (0 – 2)	1.17 ± 5.69 (0 – 38)	0	1.19 ± 5. 34 (0 – 30)	
**Belo Horizonte (N = 52)**	20.45 ± 12.64 (0 – 45)	1.64 ± 4.27 (0 – 14)	0	0	5.55 ± 11.76 (0 – 38)	0	3.45 ± 9.05 (0 – 38)	
**Curitiba (N = 12)**	7.42 ± 9.10 (0 – 25)	0.17 ± 0.58 (0 – 2)	0	1.33 ± 4.31 (0 – 15)	0	6.58 ± 22.49 (0 – 78)	2.00 ± 6.93 (0 – 24)	
**Porto Alegre (N = 15)**	4.93 ± 8.19 (0 – 33)	68.33 ± 143.40 (0 – 568)	0	0.20 ± 0.56 (0 – 2)	0	0	0	
**Mamanguape (N = 24)**	9.08 ± 18.17 (0 – 89)	65.04 ± 91.36 (0 – 399)	0.88 ± 3.88 (0 – 19)	0.38 ± 1.17 (0 – 5)	0	0	0	
**Santa Rita (N = 17)**	15.65 ± 32.56 (0 – 135)	181. 59 ± 121.09 (0 – 445)	5.06 ± 20.86 (0 – 86)	0	0	0	0	
**Guaíra (N = 14)**	10.14 ± 10.01 (0 – 28)	0	0	0	0	0	0	

N = number of hosts examined.

**Table 4:** Mean abundance of helminths ± standard deviations and range size in parenthesis in relation to host sex and age recovered from *Didelphis albiventris* by locality in Porto Alegre, Curitiba, Guaíra, Belo Horizonte, Mamanguape and Santa Rita, Brazil.

Parameters/Species	*Rhopalias horridus*	Cestoda	*Oligacanthorhynchus microcephalus*
**Mean Abundance Total (N =93)**	2.39 ± 12.45 (0 – 97)	0.05 ± 0.52 (0 – 5)	0.52 ± 2.05 (0 – 14)
**Male (N = 56)**	3.45 ± 15.54 (0 – 97)	0.09 ± 0.86 (0 – 5)	0.52 ± 2.90 (0 – 14)
**Female (N = 34)**	0.85 ± 4.47 (0 – 26)	0	0.56 ± 1.71 (0 – 8)
**Young (N = 36)**	0	0.14 ± 0.83 (0 – 5)	0
**Adult (N = 52)**	4.27 ± 16.48 (0 – 97)	0	0.92 ± 2.68 (0 – 14)
**Belo Horizonte (N = 11)**	13.82 ± 32.15 (0 – 97)	0	0
**Curitiba (N =12)**	2.17 ± 7.51 (0 – 26)	0	0
**Porto Alegre (N = 15)**	2.93 ± 10.56 (0 – 41)	0	0
**Mamanguape (N = 24)**	0	0.21 ± 1.04 (0 – 5)	0
**Santa Rita (N = 17)**	0	0	0
**Guaíra (N = 15)**	0	0	3.43 ± 4.35 (0 – 14)

N = number of hosts examined.

**Table 5:** Mean intensity of helminths ± standard deviations and range size in parenthesis in relation to host sex and age recovered from *Didelphis albiventris* by locality in Porto Alegre, Curitiba, Guaíra, Belo Horizonte, Mamanguape and Santa Rita, Brazil.

**Parameters/Species**	***Aspidodera raillieti***	***Cruzia tentaculata***	***Travassostrongylus orloffi***	***Travassostrongylus sextus***	***Trichuris didelphis***	***Trichuris minuta***	***Trichuris* sp.**
**Mean Intensity Total (N = 93)**	41.40 ± 61.97 (1 – 401)	211.92 ± 233.63 (3 – 1.125)	9.14 ± 7.22 (1 – 28)	5.00	13.92 ± 15.66 (1 – 60)	13.53 ± 17.46 (1 – 60)	2.50 ± 2.12 (1 – 4)
**Male (N = 56)**	38.87 ± 72.46 (1 – 401)	157.62 ± 172.88 (1 – 685)	6.88 ± 5.28 (1 – 15)	5.00	17.60 ± 25.07 (1 – 60)	20.33 ± 1.7.21 (2 – 41)	2.50 ± 2.12 (1 – 4)
**Female (N = 34)**	43.32 ± 42.63 (1 – 197)	290.36 ± 288.35 (8 – 1.125)	12.00 ± 8.76 (4 – 28)	0	9.71 ± 6.37 (2 – 19)	.9.82 ± 17.23. (1 – 60)	0
**Young (N = 36)**	18.63 ± 15.08 (1 – 71)	114.21 ± 139.68 (1 – 617)	16.14 ± 16.76 (4 – 50)	5.00	14.89 ± 18.12 (2 – 60)	.10.17 ± 13.77 (1 – 34)	0
**Adult (N = 52)**	52.67 ± 21.35 (1 – 401)	301.61 ± 273.32 (1 – 1.125)	7.50 ± 6.35 (1 – 16)	0	8.25 ± 5.62 (1 – 14)	.15.25 ± 11.41 (1 – 60)	2.50 ± 2.12 (1 – 4)
**Belo Horizonte (N = 11)**	20.86 ± 46.87 (1 – 127)	213.67 ± 250.67 (1 – 590)	0	0	0	0	2.50 ± 2.12 (1 – 4)
**Curitiba (N = 12)**	31.90 ± 26.26 (1 – 91)	52.09 ± 53.48 (1 – 119)	2.50 ± 2.12 (1 – 4)	5.00	0	3.00	0
**Porto Alegre (N = 15)**	32.09 ± 27.02 (4 – 71)	246.73 ± 156.11 (24 – 531)	10.67 ± 4.62 (8 – 16)	0	7.00 ± 6.00 (1 – 13)	3.40 ± 4.83 (1 – 12)	0
**Mamanguape (N = 24)**	26.33 ± 38.40 (1 – 125)	105.00 ± 123.94 (3 – 523)	7.00 ± 4.64 (1 – 13)	0	8.00 ± 8.15 (2 – 21)	4.67 ± 4.04 (1 – 9)	0
**Santa Rita (N = 17)**	79.93 ± 103.55 (7 – 401)	445.38 ± 311.84 (18 – 1.125)	20.50 ± 7.52 (8 – 28)	0	23.00 ± 25.63 (2 – 60)	.15.63 ± 20.46. (2 – 60)	0
**Guaíra (N = 14)**	76.00	118.25 ± 80.93 (99 – 250)	0	0	14.00	0	0

N = number of hosts examined.

**Table 6:** Mean intensity of helminths ± standard deviations and range size in parenthesis in relation to host sex and age recovered from *Didelphis albiventris* by locality in Porto Alegre, Curitiba, Guaíra, Belo Horizonte, Mamanguape and Santa Rita, Brazil.

**Parameters/Species**	***Turgida turgida***	***Viannaia hamata***	***Viannaia* sp.**	***Brachylaima advena***	***Duboisiella proloba***	***Plagiorchis didelphidis***	***Rhopalias coronatus***	
**Mean Intensity Total (N =93)**	13.73 ± 19.73 (1 -135)	109.36 ± 128.65 (2 – 568)	10.50 ± 12.02 (2 – 19)	4.00 ± 5.07 (1 – 15)	20.33 ± 35.15 (9 – 38)	39.50 ± 54.45 (1 – 79)	15.50 ± 13.72 (1 – 30)
**Male (N= 56)**	11.26 ± 10.88 (1 – 45)	88.45 ± 97.85 (2 – 399)	35.67 ± 44.41 (2 – 86)	1.50 ± 1.00 (1 – 3)	20.33 ± 15.50 (9 – 38)	0	12.67 ± 15.31 (1 – 30)
**Female (N = 34)**	19.14 ± 29.10 (1 – 135)	139.95 ± 163.81 (2 – 568)	0	7.33 ± 6.81 (2 – 15)	0	1.00	24.00
**Young (N = 36)**	5.46 ± 5.04 (1 – 22)	87.42 ± 82.72 (2 – 330)	2.00	6.00 ± 3.22 (1 – 15)	0	78.00	0
**Adult (N = 52)**	17.35 ± 21.35 (1 – 135)	130.81 ± 159. 87 (4 – 445)	.52.50 ± 47.38 (19 – 86)	1.33 ± 0.58 (1 – 2)	20.33 ± 15.50 (9 – 38)	0	15.50 ± 13.72 (1 – 3 0)
**Belo Horizonte (N = 52)**	20.45 ± 12.64 (1 – 45)	9.00 ± 7.07 (4 – 14)	0	0	20.33 ± 15.50 (9 – 38)	0	19.00 ± 15.31 (1 – 30)
**Curitiba (N = 12)**	11.13 ± 9.11 (1 – 25)	2.00	0	8.00 ± 9.90 (1 – 15)	0	39.50 ± 54.45 (1 – 78)	24.00
**Porto Alegre (N = 15)**	6.17 ± 8.78 (1 – 33)	85.42 ± 156.78 (4 – 568)	0	1.50 ± 0.71 (1 – 2)	0	0	0
**Mamanguape (N = 24)**	11.47 ± 19.83 (1 – 89)	74.33 ± 94.20 (2 – 399)	10.50 ± 12.02 (2 – 19)	3.00 ± 2.00 (1 – 5)	0	0	0
**Santa Rita (N = 17)**	16.53 ± 33.24 (1 – 135)	192.94 ± 106.84 (45 – 445)	86.00	0	0	0	0
**Guaíra (N = 14)**	12.91 ± 9.53 (4 – 28)	0	0	0	0	0	0

N = number of hosts examined.

**Table 7:** Mean intensity of helminths ± standard deviations and range size in parenthesis in relation to host sex and age recovered from *Didelphis albiventris* by locality in Porto Alegre, Curitiba, Guaíra, Belo Horizonte, Mamanguape and Santa Rita, Brazil.

**Parameters/Species**	***Rhopalias horridus***	**Cestoda**	***Oligacanthorhynchus microcephalus***
**Mean Intensity Total (N =93)**	44.40 ± 35.15 (3 – 97)	5.00	6.00 ± 4.17 (1 – 14)
**Male (N = 56)**	64.33 ± 29.14 (55 – 97)	5.00	7.25 ± 5.56 (1 – 14)
**Female (N = 34)**	14.50 ± 16.26 (0 – 26)	0	4.75 ± 2.36 (3 – 8)
**Young (N = 36)**	0	5.00	0
**Adult (N = 52)**	44.40 ± 30.55 (3 – 97)	0	6.00 ± 4.17 (1 – 14)
**Belo Horizonte (N = 11)**	76.00 ± 29.70 (55 – 97)	0	0
**Curitiba (N =12)**	26.00	0	0
**Porto Alegre (N = 15)**	22.00 ± 26.87 (3 – 41)	0	0
**Mamanguape (N = 24)**	0	5.00	0
**Santa Rita (N = 17)**	0	0	0
**Guaíra (N = 15)**	0	0	6.00 ± 4.49 (1 – 14)

**Table 8:** Prevalence (95% confidence interval) of helminths in relation to host sex and age recovered from *Didelphis albiventris* by locality in Porto Alegre, Curitiba, Guaíra, Belo Horizonte, Mamanguape and Santa Rita, Brazil.

**Parameters/Species**	***Aspidodera raillieti***	***Cruzia tentaculata***	***Travassostrongylus orloffi***	***Travassostrongylus sextus***	***Trichuris didelphis***	***Trichuris minuta***	***Trichuris* sp.**
**Prevalence Total (N = 93)**	66.67 (56.13 – 76.11)	81.72 (72.35 – 88.98)	15.05 (8.48 – 23.97)	1.08 (0.03 – 5.85)	12.90 (6.85 – 21.45)	18.28 (11.02 – 27.65)	2.15 (0.26 – 7.55)
**Male (N = 56)**	67.86 (57.04 – 79.72)	80.36 (67.57 – 89.77)	14.29 (6.38 – 26.22)	1.29 (0.04 – 9.55)	8.93 (2.96 – 19.62)	10.71 (4.03 – 21.88)	3.57 (0.44 – 12.31)
**Female (N = 34)**	64.71 (46.49 – 80.25)	82.35 (65.47 – 93.24)	17.65 (6.76 – 34.53)	0	20.59 (8.70 – 37.90)	32.35 (17.39 – 50.53)	0
**Young (N = 36)**	75.00 (57.80 – 87.88)	91.67 (77.53 – 98.25)	19.44 (8.19 – 36.02)	2.78 (0.07 – 14.53)	25.00 (12.12 – 42.20)	16.67 (6.37 – 32.81)	0
**Adult (N = 52)**	57.69 (43.20 – 71.27)	73.08 (58.98 – 84.43)	7.69 (2.14 – 18.54)	0	7.69 (2.14 – 18.54)	15.38 (6.88 – 28.08)	3.85 (0.47 – 13.21)
**Belo Horizonte (N = 11)**	63.64 (30.79 – 89.07)	81.82 (48.22 – 97.72)	0	0	0	0	18.18 (2.28 – 51.78)
**Curitiba (N = 12)**	83.33 (51.59 – 97.91)	91.67 (61.52 – 99.79)	16.67 (2.09 – 48.41)	8.33 (0.21 – 38.48)	0	8.33 (0.21 – 38.48)	0
**Porto Alegre (N = 15)**	73.33 (44.90 – 92.21)	100.00 (78.20 – 100.00)	20.00 (4.33 – 48.09)	0	20.00 (4.33 – 48.09)	33.33 (11.82 – 61.62)	0
**Mamanguape (N = 24)**	75.00 (53.29 – 90.23)	91.67 (73.00 – 98.97)	20.83 (7.13 – 42.15)	0	20.83 (7.13 – 42.15)	12.50 (2.66 – 32.36)	0
**Santa Rita (N = 17)**	88.24 (63.56 – 98.54)	94.12 (71.31 – 99.85)	23.53 (6.81 – 49.90)	0	23.53 (6.81 – 49.90)	47.06 (22.98 – 72.19)	0
**Guaíra (N = 14)**	7.14 (0.18 – 33.87)	28.57 (8.39 – 58.10)	0	0	7.14 (0.18 – 33.87)	0	0

N = number of hosts examined.

**Table 9:** Prevalence (95% confidence interval) of helminths in relation to host sex and age recovered from *Didelphis albiventris* by locality in Porto Alegre, Curitiba, Guaíra, Belo Horizonte, Mamanguape and Santa Rita, Brazil.

**Parameters/Species**	***Turgida turgida***	***Viannaia hamata***	***Viannaia* sp.**	***Brachylaima advena***	***Duboisiella proloba***	***Plagiorchis didelphidis***	***Rhopalias coronatus***	
**Prevalence Total (N =93)**	83.87 (74.80 – 90.68)	56.99 (46.31 – 67.22)	.3.23 (0.67 – 9.14.)	7.53 (3.08 – 14.89)	3.23 (0.67 – 9.14)	2.15 (0.26 – 7.55)	4.30 (1.18 – 10.65)
**Male (N= 56)**	82.14 (69.60 – 91.09)	55.36 (41.47 – 68.66)	5.36 (1.12 – 14.87)	7.14 (1.98 – 17.29)	5.36 (1.12 – 14.87)	0	5.36 (1.12 – 14.87)
**Female (N = 34)**	82.35 (65.47 – 93.24)	58.82 (40.70 – 75.35)	0	8.82 (1.86 – 23.68)	0	2.94 (0.07 – 15.33)	2.94 (0.07 – 15.33)
**Young (N = 36)**	77.78 (60.85 – 89.88)	72.22 (54.81 – 85.80)	2.78 (0.07 – 14.53)	11.11 (3.11 – 26.06)	0	2.78 (0.07 – 14.53)	0
**Adult (N = 52)**	2.78 (0.07 – 14.53)	40.38 (27.01 – 54.90)	.3.85 (0.47 – 13.21.)	5.77 (1.21 – 15.95)	5.77 (1.21 – 15.95)	0	7.69 (2.14 – 18.54)
**Belo Horizonte (N = 52)**	100.00 (71.51 – 100.00)	18.18 (2.28 – 51.78)	0	0	27.27 (6.02 – 60.97)	0	18.18 (2.28 – 51.78)
**Curitiba (N = 12)**	66.67 (34.89 – 51.78)	8.33 (0.21 – 38.48)	0	16.67 (2.09 – 48.41)	0	16.67 (2.09 – 48.41)	8.33 (0.21 – 38.48)
**Porto Alegre (N = 15)**	80.00 (51.91 – 95.67)	80.00 (51.91 – 95.67)	0	13.33 (1.66 – 40.46)	0	0	0
**Mamanguape (N = 24)**	79.17 (57.85 – 92.87)	87.50 (67.64 – 97.34)	8.33 (1.03 – 27.00)	12.50 (2.66 – 32.36)	0	0	0
**Santa Rita (N = 17)**	94.12 (71.31 – 99.85)	94.12 (71.31 – 99.85)	5.88(0.15 – 28.69)	0	0	0	0
**Guaíra (N = 14)**	78.57 (49.20 – 95.34)	0	0	0	0	0	0

N = number of hosts examined.

**Table 10:** Prevalence (95% confidence interval) of helminths in relation to host sex and age recovered from *Didelphis albiventris* by locality in Porto Alegre, Curitiba, Guaíra, Belo Horizonte, Mamanguape and Santa Rita, Brazil.

**Parameters/Species**	***Rhopalias horridus***	**Cestoda**	***Oligacanthorhynchus microcephalus***
**Prevalence Total (N =93)**	4.30 (1.18 – 10.65)	1.08 (0.03 – 5.85)	8.60 (3.79 – 16.25)
**Male (N = 56)**	5.36 (1.12 – 14.87)	1.79 (0.04 – 9.55)	7.14 (1.98 – 17.29)
**Female (N = 34)**	5.88 (0.72 – 19.68)	0	11.76 (3.30 – 27.45)
**Young (N = 36)**	0	2.78 (0.07 – 14.53)	0
**Adult (N = 52)**	9.62 (3.20 – 21.03)	0	15.38 (6.88 – 28.08)
**Belo Horizonte (N = 11)**	18.18 (2.28 – 51.78)	0	0
**Curitiba (N =12)**	8.33 (0.21 – 38.48)	0	0
**Porto Alegre (N = 15)**	13.33 (1.66 – 40.46)	0	0
**Mamanguape (N = 24)**	0	4.17 (0.11 – 21.12)	0
**Santa Rita (N = 17)**	0	0	0
**Guaíra (N = 15)**	0	0	57.14 (28.86 – 82.34)

N = number of hosts examined.

In Porto Alegre, *C. tentaculata* and *V. hamata* had the highest mean abundances (231.31 ± 162.95 and 68.33 ± 143.40, respectively) (Tables 2, 3 and 4) and mean intensities (246. 73 ± 156.11 and 85.42 ± 156.78, respectively) (Tables 5, 6 and 7). In Curitiba, *C. tentaculata*, *A. raillieti*, and *Tu. turgida* were the most prevalent species (91.67 [61.52 – 99.79], 83.33 [51.59 – 97.91], and 66.67 [34.89 – 51.78], respectively) (Tables 8, 9 and 10). In Guaíra, *C. tentaculata* and *A. raillieti* had the highest mean intensities (118.25 ± 80.93 and 76.00, respectively) (Tables 5, 6 and 7), and *Tu. turgida* had the highest prevalence (78.57 [49.20 – 95.34]) (Tables 8, 9 and 10). In Belo Horizonte, *C. tentaculata* had the highest mean abundance (174.82 ± 240.29) (Tables 2, 3 and 4) and mean intensity (213.67 ± 250.67) (Tables 5, 6 and 7). *Tu. turgida* had the highest prevalence (100.00 [71.51 – 100.00]) (Tables 8, 9 and 10). In Mamanguape, *C. tentaculata* and *V. hamata* had the highest abundances (96.25 ± 121.22 and 65.04 ± 91.36, respectively) (Tables 2, 3 and 4). *C. tentaculata*, *A. raillieti*, *Tu. turgida* and *V. hamata* had the highest prevalence rates (91.67 [73.00 – 98.97], 75.00 [53.29 – 90.23], 79.17 [57.85 – 92.87] and 87.50 [67.64 – 97.34], respectively) (Tables 8, 9 and 10). In Santa Rita, *C. tentaculata*, *V. hamata* and *A. raillieti* had the highest abundances (419.18 ± 320.69, 181.59 ± 121.09 and 70.53 ± 100.44, respectively) (Tables 2, 3 and 4), and *C. tentaculata* and *V. hamata* had the highest mean intensities (445.38 ± 311.84 and 192.94 ± 106.84, respectively) (Tables 5, 6 and 7).

In relation to the host sex for the set of locations, *A. raillieti* and *R. horridus* mostly occurred in male hosts, and *Viannaia* sp. *Trichuris* sp., *T. sextus*, *D. proloba* and Cestoda occurred only in male hosts. *C. tentaculata. Tu. turgida, Tr. didelphis, Tr. minuta*, *V. hamata, T. orloffi, B. advena, P. didelphidis, R. coronatus and O. microcephalus* mostly occurred in female hosts. The sex of three hosts was not recorded, one in Curitiba, one in Porto Alegre and one in Santa Rita. With respect to host age, the species *C. tentaculata, A. raillieti*, *Tu. turgida, Tr. minuta* and *Viannaia* sp. mostly occurred in adult hosts. *Trichuris* sp., *R. horridus, R. coronatus, O. microcephalus* and *D. proloba* occurred only in adult hosts. The species *Tr. didelphis*, *V. hamata, T. orloffi*, *and B. advena* mostly occurred in young hosts. *Travassostrongylus sextus* and Cestoda occurred only in young hosts.

The GLM results indicated that all the tested predictors influenced helminth species richness, with a greater effect of locality, which was present in all the models considered plausible, and the type of land use, host sex and age, which were present in four plausible models each (Table 11). Land use had a positive effect on species richness, indicating higher helminth richness in preserved areas than in peri-urban areas (Table 1). Host sex and age were also present in the selected models. Greater species richness was associated with female host and adult host specimens (Tables 2, 3 and 4).

**Table 11:** Results of the Generalized Linear Models (GLM) for the helminth species richness recovered from *Didelphis albiventris* in Porto Alegre, Curitiba, Guaíra, Belo Horizonte, Mamanguape and Santa Rita, Brazil, in relation to host age (young or adult) and sex (male or female), locality, and type of land use.

**Models**	**AICc**	**ΔAICc**	**AICcWt**	**K**
Locality	312.7	0	0.178	7
Locality + Land use	312.7	0	0.178	7
Age + Locality	313.3	0.58	0.133	8
Age + Locality + Land use	313.3	0.58	0.133	8
Locality + Sex	313.6	0.94	0.112	8
Locality + Sex + Land use	313.6	0.94	0.112	8
Age + Locality + Sex	314.4	1.69	0.077	9
Age + Locality + Sex + Land use	314.4	1.69	0.077	9
Sex + Land use	334.2	21.55	0	4
Land use	335	22.27	0	3
Age + Sex + Land use	336.4	23.73	0	5
Age + Land use	337.1	24.37	0	4
Null	348	35.29	0	2
Age	348.1	35.37	0	3
Age + Sex	349.1	36.36	0	4
Sex	349.3	36.57	0	3

AICc = corrected Akaike information criterion; ΔAICc = difference between the AICc of a model and the model with the lowest AICc; AICcWt = Akaike weights; K = number of model parameters.

Similarly to species richness, all the tested predictors were indicated to have influenced helminth abundance, and a greater importance was observed for locality and host age, which were present in all the plausible models (Table 12). Helminth abundance was significantly greater among adult hosts, female hosts, and those from preserved environments (Tables 2, 3 and 4).

**Table 12:** Results of the Generalized Linear Models (GLM) for the helminth abundance recovered from *Didelphis albiventris* in Porto Alegre, Curitiba, Guaíra, Belo Horizonte, Mamanguape, and Santa Rita, Brazil, in relation to host age (young or adult) and sex (male or female), locality, and type of land use.

**Models**	**AICc**	**ΔAICc**	**AICcWt**	**K**
Age + Sex + Locality	1260.6	0	0.288	9
Age + Sex + Locality + Land use	1260.6	0	0.288	9
Age + Locality	1261.2	0.61	0.212	8
Age + Locality + Land use	1261.2	0.61	0.212	8
Locality + Sex	1273.9	13.37	0	8
Locality + Sex + Land use	1273.9	13.37	0	8
Locality	1274.7	14.1	0	7
Locality + Land use	1274.7	14.1	0	7
Age + Sex + Land use	1295	35.24	0	5
Age + Land use	1301.5	40.94	0	4
Sex + Land use	1310.2	49.67	0	4
Land use	1315.4	54.79	0	3
Age + Sex	1323.8	63.28	0	4
Sex	1324.1	64.21	0	3
Age	1324.8	64.21	0	3
Null	1325.6	65	0	2

AICc = corrected Akaike information criterion; ΔAICc = difference between the AICc of a model and the model with the lowest AICc; AICcWt = Akaike weights; K = number of model parameters.

Regarding the effect of spatial scale on helminth abundance, we found a significant association with the first dbMEM axis (dbMEM1; F = 12.67; df = 1; p = 0.001). This result indicates that the abundance patterns of helminth infracommunities from geographically distant hosts differ along the spatial gradient.

## Discussion

Among the 14 helminth species identified, 13 had previously been reported to parasitize *Didelphis albiventris.* This opossum was recently reported as a new host for *Travassostrongylus sextus* (Cirino et al., 2025). Curitiba is a new locality of occurrence for the nematodes *A. raillieti, C. tentaculata, T. turgida, T. orloffi, T. sextus*, and *V. hamata* and for the trematodes *B. advena, P. didelphidis, R. coronatus, and R. horridus.* Guaíra has its first record for the nematodes *A. raillieti*, *C. tentaculata*, *T. turgida*, *T. didelphis,* and for the acanthocephalan *O. microcephalus.* Belo Horizonte represents a new distribution area for the trematodes *R. horridus* and *D. proloba*. Santa Rita represents a new area of occurrence for the nematodes *A. raillieti*, *C. tentaculata*, *T. turgida*, *T. didelphis, Tr. minuta, V. hamata*, and *T. orloffi.*

Compared with the other species, *C. tentaculata*, *A. raillieti*, and *T. turgida* had the highest abundance and prevalence, and *C. tentaculata* had the highest mean intensity. Quintão e Silva & Costa (1999) reported a relatively high prevalence of these three species and a high intensity of *C. tentaculata* in *D. albiventris* helminth communities in the region of Pampulha (Belo Horizonte, MG). Higher intensities were recorded for *C. tentaculata* and *V. hamata,* corroborating the results reported by Müller (2005) for the same host in the municipality of Pelotas, RS. Zabott et al. (2017) reported a relatively high prevalence of *C. tentaculata* and *T. turgida* in *D. albiventris* compared with other helminths collected in the municipality of Palotina, PR. Cirino et al. (2022) reported a high prevalence of these three nematode species in *D. albiventris*. In the study by Hartmann et al. (2025), *C. tentaculata* was the most abundant and prevalent species in *D. abiventris* in Argentina.

*Cruzia tentaculata*, *A. raillieti*, and *T. turgida* are frequently reported in didelphids in different environments, whose abundance, prevalence and/or intensity are usually high. Acosta-Virgen et al. (2015) recorded these three species parasitizing *Didelphis virginiana* and *Didelphis marsupialis* in Mexico; Chero et al. (2017) reported on *D. marsupialis* in Peru; Costa-Neto et al. (2019) and Boullosa et al. (2021) reported on *D. aurita* in the state of Rio de Janeiro, Brazil; Freitas et al. (2022) reported on *D. marsupialis* in the state of Mato Grosso, Brazil; and Hartmann et al. (2025) reported on *D. albiventris* and *D. aurita* in Argentina. The relatively high occurrence and wide geographic distribution of *C. tentaculata*, *A. raillieti*, and *T. turgida* indicate that these species act as central components in the structure of the helminth communities of marsupials of the genus *Didelphis*. (Costa-Neto et al., 2019; Cirino et al., 2022). Thus, *C. tentaculata*, *A. raillieti*, and *T. turgida* can be considered *core* species of the helminth fauna of *D. albiventris*. In addition, *D. albiventris* can occur in sympatry with *D. aurita* in areas of the Atlantic Forest (see Figure 1B), where they share the same habitat and resources. In these regions, both species exhibit very similar helminth communities, suggesting that parasite sharing is facilitated by niche overlap and exposure to common sources of infection (Hartmann et al., 2025).

With respect to intrinsic factors, female hosts recorded greater helminth species richness and abundance than males did, a result that contradicts expectations, since male specimens of mammals tend to have a larger home range than females do and, consequently, greater exposure to sources of infection in the environment (Zuk & McKean, 1996; Klein, 2004). However, we must mention that the analysis included all the localities and that we captured more male hosts.

The age of the hosts influenced helminth species richness and abundance, indicating higher levels of infection in adult hosts than in young individuals, as expected. Compared with young individuals, adult mammalian hosts can accumulate more parasites throughout life, increasing their infection rates (Behnke et al., 1999; Poulin, 2013). Similar results were reported by Illia et al. (2024), who reported that young specimens of *D. albiventris* were less parasitized than adult individuals were. In general, these animals do not clear helminth infections over time because the immunity acquired against helminths is, in most cases, partial and not very effective due to the helminth ability to modulate the host's immune system and, therefore, enable long-term persistence within a host and reinfection (Combes, 2001).

With respect to extrinsic factors, the GLM results revealed that helminth species richness and abundance may be influenced by locality, suggesting that differences between localities were determinants of parasite diversity in *D. albiventris*. Local conditions, such as sunlight incidence and humidity, may influence the community structure of helminths (Bordes et al., 2009; Cardoso et al., 2016). The type of land use also seemed to be an important predictor of helminth species richness and abundance, with higher values observed in preserved areas than in peri-urban areas, indicating that more preserved environments may favor a greater diversity of parasites, a finding that has been reported in other studies (Cardoso et al., 2016; Costa-Neto et al., 2019; Cirino et al., 2022). These results support the hypothesis that fragmentation and anthropogenic disturbance reduce the diversity and transmission of parasites, possibly because of the loss of intermediate hosts and the alteration of the microhabitats necessary for the life cycles of helminths (Cardoso et al., 2016; Kiene et al., 2021). Indeed, intermediate hosts can be considered sources of parasites for definitive hosts (Poulin, 2007). In addition, approximately half of the helminth species recorded in this study have an indirect life cycle. The negative effects of fragmentation on the life cycle of parasites have been reported in several studies (Lafferty & Kuris 1999; Patz et al., 2000; Pakdeenarong et al., 2014).

The influence of spatial scale (dbMEM 1 axis) on helminth abundances among infracommunities suggests that the movement of hosts and their spatial dynamics allowed homogenization in terms of helminth species composition and abundance at the local scale, with greater sharing of parasite species among geographically close infracommunities. This pattern may be explained by the ecological traits of opossums that occupy several environments, have generalist habits, and have a strong ability to adapt to disturbed environments (Gentile et al., 2018). In addition, by maintaining large home ranges to secure essential resources, these hosts are more frequently exposed to parasite transmission (Cáceres, 2012). In general, parasite communities that are geographically distant from each other tend to have different species and/or parasite loads because of changes in landscape structure, environmental conditions and the availability of definitive and intermediate hosts (Poulin, 2003; Krasnov et al., 2005). However, local conditions and the level of disturbance in the environment may also influence parasite fauna across areas (Lafferty & Kuris, 1999; Cardoso et al., 2016).

Given the observational design of this study, we highlight that the reported relationships found should be interpreted with caution. The data were collected during different periods and seasons of the year for each locality, without temporal replication, which may represent a limitation in the interpretation of the results because of the likely unmeasured effect of seasonality on the parasitism of this host. In addition, other factors such as climate, habitat structure or intrinsic factors of the parasites, which were not analyzed in this study, might also have influenced the helminth structure observed.

## Conclusions

The helminth species richness and abundance of *D. albiventris* were influenced by both factors intrinsic to the host (sex and age) and factors extrinsic to the host (locality and land use), suggesting that several factors can act in an integrated way in host‒parasite interactions in wildlife. The spatial scale seemed to influence the structure of the helminth infracommunities of *D. albiventris,* with greater differences with increasing geographical distance, suggesting that the helminth communities of the infracommunities of this host are structured as a function of local characteristics of the environment, although there are cross sectional patterns in structuring the communities, such as host characteristics.

## Data Availability

Data related to the study are included in the article.
